# Acetaminophen-induced Hepato- and Nephrotoxicity and Amelioration by Silymarin and *Terminalia chebula* in Rats

**DOI:** 10.4103/0971-6580.72672

**Published:** 2010

**Authors:** K. S. Gopi, A. Gopala Reddy, K. Jyothi, B. Anil Kumar

**Affiliations:** Department of Pharmacology and Toxicology, College of Veterinary Science, Hyderabad - 500030, Andhra Pradesh, India

**Keywords:** Acetaminophen, hepatotoxicity, nephrotoxicity, silymarin, *Terminalia chebula*

## Abstract

Experimental study was conducted to evaluate the hepato- and renoprotective effect of silymarin and *Terminalia chebula* against experimentally-induced acetaminophen (APAP) toxicity in rats. Oral administration of APAP @ 500 mg/kg for 1 to 3 days to all the four groups (six rats in each) resulted in significant elevation of serum triglycerides, total cholesterol, blood urea nitrogen, serum creatinine, and aspartate transaminase activity. Post-treatment with silymarin @ 25 mg/kg and *T. chebula* 125 mg/kg in groups 2 and 3 and their combination to group 4 from day 4 to 14 has significantly reversed the alterations of above said markers and offered better protection. The results of the study enunciated that silymarin and *T. chebula* exhibit good hepato- and nephro-protection against APAP toxicity.

## INTRODUCTION

Liver diseases remain one of the serious health problems. Hepatic dysfunction due to ingestion or inhalation of hepatotoxin is increasing worldwide.[1] Acetaminophen (APAP; paracetamol-[PCM]) is commonly prescribed analgesic and antipyretic drug producing dose-dependent hepato- and nephrotoxicity. Herbal alternatives are one of the best ways to minimize liver damage and were used prophylactically and also as antidotes. Silymarin, a flavonolignan obtained from *Silybum marianum*, comprises of silybin as the most active component that counteracts the leakage of alanine transaminase (ALT) and gamma glutamyl transferase and prevent lipid peroxidation (LPO).[2] The fruits of *Terminalia chebula* were used as common household remedy,[3] and its phenolics prevent nickel chloride-induced oxidative stress by decreasing LPO, restoring the activity of glutathione-S-transferase, glutathione reductase, and glutathione peroxidase.[4] The present investigation was carried out to study the hepato- and renoprotective effects of silymarin and *T. chebula*.

## MATERIALS AND METHODS

A total of 24 male *Wistar kyoto* rats (200 – 250 g) were obtained from National Institute of Nutrition, Hyderabad. The animals were maintained and used in accordance with guideline of the Control and Prevention Supervision Committee Experimental on Animals, and the protocols were accepted by Instituitional animal ethics committee (IAEC). The rats were randomly divided into four groups of six rats in each; APAP @ 500 mg/kg was administered to all the groups from 1 to 3 days. Group 1 served as toxic control and was maintained with distilled water from 4 to 14 days. Groups 2 and 3 were administered orally with silymarin (25 mg/kg) and *T. chebula* (125 mg/kg) from 4 to 14 days. Group 4 was administered with a combination of silymarin + *T. chebula* at the doses mentioned in groups 2 and 3, respectively. The experiment was carried out for 14 days.

The sera samples were separated from blood on day 0, 4, and 14 for the estimation of triglycerides, total cholesterol, blood urea nitrogen (BUN), serum creatinine, and aspartate transaminase (AST) activity by using commercially available diagnostic kits (Qualigens Pvt. Ltd, Mumbai, India). All data were expressed as means ± SE. The means were analyzed by One Way Analysis of Variance (ANOVA) by using SPSS software package. Values of *P*<0.05 were considered as significant.

## RESULTS AND DISCUSSION

The activity of AST (U/L) [Table 1] and concentration of triglycerides (mg/dl) and total cholesterol (mg/dl) [Table 2] were significantly (*P*<0.05) elevated on day 4 in groups 1, 2, 3, and 4 as compared with the mean values of day 0, and these observations coincided with earlier studies where oral administration of PCM at 750 mg/kg/day for 7 days increased the level of liver marker enzymes *viz*., AST, ALT, Alkaline phosphatase (ALP), creatinine, and BUN.[5] Following administration with silymarin and *T. chebula* in groups 2, 3, and 4, the levels were significantly (*P*<0.05) reduced on day 14 as compared with that of hepatotoxic control.

**Table 1:** Aspartate transaminase activity (U/l) in different groups of rats

Group	Day		
	0	4	14
Acetaminophen (APAP) (1 – 3 days)	71.00 ± 3.07^aA^	127.65 ± 8.90^aBC^	119.78 ± 4.23^bB^
APAP (1 – 3 days) + silymarin (4 – 14 days)	67.71 ± 3.40^aA^	124.65 ± 8.62^aBC^	81.57 ± 3.33^aA^
APAP (1 – 3 days) + *Terminalia chebula* (4 – 14 days)	69.94 ± 6.21^aA^	140.94 ± 5.78^aBC^	74.01 ± 8.85^aA^
APAP (1 – 3 days) + silymarin + *Terminalia chebula* (4 – 14 days)	74.20 ± 5.16^aA^	130.56 ± 8.31^aBC^	83.42 ± 4.76^aA^

Values are mean ± SE of six observations; Means with different alphabets as superscripts differ significantly (*P*<0.05) ANOVA; Capital alphabets (horizontal comparison), small alphabets (vertical comparison); SE - Standard error

**Table 2:** Serum triglycerides and total cholesterol concentration in different groups of rats

Group	Serum triglycerides concentration (mg/dl)			Serum total cholesterol concentration (mg/dl)		
	Day 0	Day 4	Day 14	Day 0	Day 4	Day 14
Acetaminophen (APAP) (1 – 3 days)	26.34 ± 2.32^aAB^	58.09 ± 4.53^aC^	53.97 ± 2.12^bC^	54.95 ± 2.97^aA^	88.83 ± 5.80^aC^	84.86 ± 2.26^bC^
APAP (1 – 3 days) + silymarin (4–14 days)	24.44 ± 2.58^aAB^	62.86 ± 2.70^aC^	30.16 ± 3.53^aAB^	56.58 ± 3.73^aA^	84.50 ± 3.99^aC^	69.55 ± 2.48^aB^
APAP (1 – 3 days) +*Terminalia chebula* (4 – 14 days)	30.47 ± 2.25^aAB^	56.83 ± 3.46^aC^	32.38 ± 2.95^aB^	55.86 ± 2.11^aA^	84.86 ± 4.95^aC^	67.93 ± 2.68^aB^
APAP (1 – 3 days) + silymarin + *Terminalia chebula* (4 – 14 days)	21.58 ± 3.86^aA^	53.65 ± 4.73^aC^	24.76 ± 2.30^aAB^	62.70 ± 2.33^aAB^	90.09 ± 4.98^aC^	71.53 ± 2.09^aB^

Values are mean ± SE of six observations; Means with different alphabets as superscripts differ significantly (*P*<0.05) ANOVA; Capital alphabets (horizontal comparison), small alphabets (vertical comparison)

In the current investigation, renal biomarkers like BUN (mg/dl) and serum creatinine (mg/dl) were significantly (*P*<0.05) elevated on day 4 in groups 1, 2, 3, and 4 [Table 3] as compared with the mean values of day 0. The N-acetyl-p-benzoquinoneimine, a reactive metabolite of APAP that causes oxidative damage to tissues, might be the reason for its renotoxic effects.[6] At the end of treatment on day 14, the BUN level was significantly (*P*<0.05) decreased in groups 2, 3, and 4. However, groups treated with silymarin (group 2) and silymarin + *T. chebula* (group 4) showed a significant (*P*<0.05) decrease in BUN level when compared with *T. chebula* alone treated group (group 3) at 14^th^ day [Table 3]. Nonprotein nitrogenous substances such as BUN and serum creatinine are increased only when renal function is below 30% of its original capacity. An increase in BUN reflects an accelerated rate of protein catabolism.[7] Cytochrome P450-mediated toxic metabolite of acetaminophen binds with cell macromolecules, which might be the reason for its nephrotoxic effects.[6] The beneficial effects of the drugs in test may be attributed to free radical scavenging activity of *T. chebula*[8] and renoprotective effects of *T. chebula*.[9] Silymarin has been reported to possess protective action on kidney cells against PCM-induced toxicity.[10]

**Table 3:** Blood urea nitrogen and serum creatinine concentration in different groups of rats

Group	Blood urea nitrogen (BUN) (mg/dl)			Serum creatinine concentration (mg/dl)		
	Day 0	Day 4	Day 14	Day 0	Day 4	Day 14
Acetaminophen (APAP) (1 – 3 days)	23.14 ± 1.85^aABC^	38.49 ± 2.65^aEF^	36.10 ± 2.57^cE^	0.77 ± 0.03^aAB^	1.42 ± 0.14^aE^	1.09 ± 0.05^bCD^
APAP (1 – 3 days) + silymarin (4 – 14 days)	18.24 ± 2.05^aA^	38.62 ± 0.81^aEF^	29.18 ± 0.77^abcD^	0.70 ± 0.06^aAB^	1.18 ± 0.10^aD^	0.88 ± 0.08^aABC^
APAP (1 – 3 days) +*Terminalia chebula* (4 – 14 days)	20.63 ± 2.97^aAB^	40.50 ± 2.45^aEF^	33.98 ± 0.73^bcDE^	0.66 ± 0.04^aA^	1.16 ± 0.06^aD^	0.90 ± 0.07^abBC^
APAP (1 – 3 days) + silymarin +*Terminalia chebula* (4 – 14 days)	20.88 ± 2.04^aAB^	44.65 ± 2.66^aF^	26.92 ± 1.87^aBC^	0.77 ± 0.03^aAB^	1.18 ± 0.06^aD^	0.85 ± 0.02^aAB^

Values are mean ± SE of six observations; Means with different alphabets as superscripts differ significantly (*P*<0.05) ANOVA; Capital alphabets (horizontal comparison), small alphabets (vertical comparison)

## References

[1] Wogan GN, Cameron HM, Linsell DH, Warwick GP (1976). The induction of liver cell cancer by chemical. Liver cell cancer.

[2] Letteron P, Labbe G, Degott C, Berson A, Fromentry B, Decaforge M (1990). Mechanism of protective effects of silymarin against carbontetrachloride-induced lipid peroxidation and hepatotoxicity in mice. Biochem Pharmacol.

[3] Kirtikar KR, Basu BD (1935). Terminalia chebula. In: Indian Medicinal Plants.

[4] Prasad L, Husain Khan T, Jahangir T, Sultana S (2006). Chemomodulatory effects of Terminalia chebula against nickel chloride induced oxidative stress and tumor promotion response in male Wistar rats. J Trace Elem Med Biol.

[5] Zaher A, Hady H, Mahmoud M, Farrag M (2008). The potential protective role of alpha-lipoic acid against acetaminophen-induced hepatic and renal damage. Toxicol.

[6] Richie JP, Long CA, Chen TS (1992). Acetaminophen-induced depletion of glutathione and cysteine in aging mouse kidney. Biochem Pharmacol.

[7] Kaneko JJ, Harvey JW, Michael LB (1997). Clinical Biochemistry of Domestic Animals.

[8] Lee HS, Won NH, Kim KH, Lee H, Jun W, Lee KW (2005). Antioxidant effects of aqueous extract of Terminalia chebula *in vivo* and in vitro. Biol Pharmaceutical Bull.

[9] Rao NK, Nammi S (2006). Antidiabetic and renoprotective effects Terminalia chebula Retz. Seeds in streptozotocin-induced diabetic rats. BMC Complement Altern Med.

[10] Deák G, Müzes G, Láng I, Nékám K, González-Cabello R, Gergely P (1990). Effects of two bioflavanoids on certain cellular immune reactions. Acta Physiol Hung.

